# HIV prevention at drug shops: awareness and attitudes among shop dispensers and young women about oral pre-exposure prophylaxis and the dapivirine ring in Shinyanga, Tanzania

**DOI:** 10.1186/s12981-021-00343-1

**Published:** 2021-04-26

**Authors:** Julia Tubert, Laura Packel, Lauren A. Hunter, Rashid Mfaume, Prosper Njau, Angela A. Ramadhani, Jenny X. Liu, Sandra I. McCoy

**Affiliations:** 1 School of Public Health, University of California, Berkeley, 2121 Berkeley Way, Berkeley, CA 94704 USA; 2 Shinyanga Regional Medical Office, Shinyanga, Tanzania; 3 Health for a Prosperous Nation, Dar es Salaam, Tanzania; 4 National AIDS Control Programme, Ministry of Health, Community Development, Gender, Elderly, and Children, Dar es Salaam, Tanzania; 5 Institute for Health and Aging, Bixby Center for Global Reproductive Health, University of California, San Francisco, San Francisco, CA USA

**Keywords:** HIV prevention, Pre-exposure prophylaxis, PrEP, Young women, Sub-Saharan Africa, HIV, Tanzania, Drug shops, Long-acting PrEP, Dapivirine ring, Adolescent girls

## Abstract

**Background:**

HIV risk remains high among adolescent girls and young women (AGYW, ages 15–24) in Tanzania. Many AGYW experience stigma and provider bias at health facilities, deterring their use of HIV prevention services. Privately-owned drug shops, ubiquitous in many communities, may be an effective and accessible channel to deliver HIV prevention products to AGYW, including oral pre-exposure prophylaxis (PrEP) and the dapivirine vaginal ring.

**Methods:**

In July–August 2019, we enrolled 26 drug shops in Shinyanga, Tanzania in an ongoing study to create “girl-friendly” drug shops where AGYW can access HIV self-testing and contraception. At baseline, all shop dispensers were given basic information about oral PrEP and the dapivirine ring and were asked about their interest in stocking each. During the next 3–5 months, we surveyed AGYW (n = 56) customers about their interest in oral PrEP and the ring.

**Results:**

Among dispensers, the median age was 42 years and 77% were female. Overall, 42% of dispensers had heard of a medication for HIV prevention. Almost all dispensers reported some interest in stocking oral PrEP (92%) and the dapivirine ring (96%). Most (85%) reported they would provide oral PrEP to AGYW who requested it. Among AGYW customers, the median age was 17 years; 29% of AGYW were married or had a steady partner and 18% had children. Only 20% of AGYW had heard of a medication to prevent HIV, yet 64% and 43% expressed some interest in using oral PrEP and the dapivirine ring, respectively, after receiving information about the products. PrEP interest was higher among AGYW who were partnered and had children.

**Conclusions:**

Despite low prior awareness of PrEP among shop dispensers and AGYW, we found high levels of interest in oral PrEP and the dapivirine ring in both groups. Community-based drug shops represent a promising strategy to make HIV prevention more accessible to AGYW.

## Background

Adolescent girls and young women (AGYW, ages 15–24) comprise 25% of new adult HIV infections in sub-Saharan Africa [1]. In many countries where pre-exposure prophylaxis (PrEP) is available, AGYW are prioritized for PrEP outreach [2], yet early evidence demonstrates suboptimal PrEP uptake and coverage among AGYW [3, 4]. The gap in implementation may be partially explained by the unique barriers AGYW face when accessing sexual and reproductive health (SRH) services in healthcare facilities, such as stigma and provider bias [5–9]. Shortages of workers and supplies also impede delivery of services at many facilities [10], forcing AGYW to travel longer distances at greater costs [11, 12]. Thus, the potential for PrEP to halt HIV transmission among AGYW hinges on identifying implementation models that can mitigate these pervasive access barriers.

Innovative models to improve access to PrEP through the private sector are beginning to emerge, albeit largely in the United States [13, 14]. These strategies range from pharmacies providing PrEP referrals to the provision of full PrEP services, such as Seattle’s One-step PrEP program and San Francisco’s Mission Wellness Pharmacy [15–19]. Along this spectrum is the distribution of PrEP starter packs by pharmacists, a model recently adopted in California [20]. While PrEP typically requires a prescription from a licensed physician, this model allows pharmacists to provide 1–2 months of pills upon proof of a negative HIV test, along with a referral for lab testing and subsequent prescriptions. While there is growing interest in implementing similar private-sector models to deliver PrEP to high-priority populations in sub-Saharan Africa, few studies have explored this potential to date [21–23].

In Tanzania, the accredited drug dispensing outlet (ADDO) program has played a key role in improving access to quality-assured pharmaceuticals via community-based retail drug shops [24–26]. Located in nearly every community, ADDOs already serve as a primary source of SRH products for AGYW and may have the potential to increase access to HIV prevention services [25, 27]. Currently, Gilead’s oral PrEP pill, Truvada, is approved in Tanzania, while the vaginal dapivirine ring and generic versions of oral PrEP are pending regulatory approval [28]. In July 2020, the European Medicines Agency adopted a positive opinion on the dapivirine ring, an important step towards making the ring available in parts of sub-Saharan Africa, potentially as early as 2021 [29]. Adding these PrEP formulations to drug shop services, as they are rolled out in Tanzania, could complement existing ADDO initiatives and provide one-stop-shopping for many AGYW. With an eye toward improving access to HIV prevention technologies for AGYW, we explored the acceptability and appropriateness of PrEP distribution via privately-owned drug shops. In particular, we assessed prior knowledge of PrEP and interest in stocking/using PrEP (both oral PrEP and the forthcoming dapivirine ring) among shop dispensers and AGYW customers, as well as dispenser willingness to provide PrEP to AGYW. Understanding knowledge and attitudes around PrEP among these stakeholders is an important step in the early exploration of ADDO-based PrEP delivery models in Tanzania.

## Methods

In July–August 2019, we recruited 26 drug shops in Shinyanga, Tanzania as part of a pilot randomized study to create “girl-friendly” drug shops where AGYW can access HIV self-testing kits and other SRH products. Shinyanga is a resource-constrained, semi-rural region with 5.1% HIV prevalence among AGYW [30]. Twenty ADDOs were randomly sampled from a government registry; three additional ADDOs and three pharmacies were purposefully sampled to increase heterogeneity in shop size and type. Drugs shops were recruited in collaboration with the Municipal Pharmacist who oversees ADDOs in Shinyanga and who introduced the research team to shop owners. Eligible shops met the following criteria: (1) ADDO or pharmacy in Shinyanga, (2) owner was at least 18 years of age, and (3) owner was interested in participating in the parent study. Data collection was conducted through December 2019.

At enrollment, we interviewed the owner or a designated staff member (hereafter, “dispenser”) at each shop about shop operations and their perceptions of SRH products. Dispensers were asked if they had heard of a medicine that can prevent HIV infection (‘yes,’ ‘no,’ or ‘unsure’). Respondents were verbally given basic information about oral PrEP and the dapivirine ring. For oral PrEP, respondents were given the following information verbally: “Pre-exposure prophylaxis (also called PrEP) is medicine that a person can take to help prevent infection from HIV. It is taken in tablet form (a pill like Panadol). When taken daily, it lowers the chance of getting infected with HIV.” Similarly, respondents received the following information, verbally, about the dapivirine ring: “The dapivirine ring is a method that women can use to help prevent infection from HIV. It is a flexible ring which women can insert in the vagina. Over the course of the month, it releases a medicine that lowers the chance of getting infected with HIV. It must be replaced each month.” Dispensers were then asked about their interest in stocking each product, if they believed their customers would be interested in purchasing each product, and if they would be willing to sell oral PrEP to AGYW. Possible responses for each of these questions were ‘yes’, ‘no’, or ‘maybe’.

Within the subsequent 3–5 months, exit interviews were conducted during 3-h time blocks as part of the parent study to survey AGYW customers upon exit about their experiences and interactions with dispensers. AGYW customers were eligible to participate if they were 15–24 years of age. AGYW who consented to participate completed the survey with a trained research assistant in the back room of the shop or another private location nearby. Like dispensers, AGYW were asked if they had heard of a medicine for HIV prevention (‘yes’, ‘no’, or ‘unsure’) and then verbally given basic information about oral PrEP and the dapivirine ring and asked if they would be interested in using each product if it became available (‘yes’, ‘no’, or ‘maybe’). Food insecurity among AGYW was measured using the Household Hunger Scale, which has been validated for cross-cultural use [31].

We also assessed bivariate associations between sociodemographic characteristics and interest in PrEP among AGYW, using Kruskal–Wallis tests for continuous variables and Fisher exact tests for categorical variables. Interest in PrEP was dichotomized as some interest (‘yes’ or ‘maybe’) in at least one of the two PrEP modalities versus no interest in either product. Due to unstable estimates and low precision resulting from small sample size, we did not report multivariable results for this study.

## Results

**Fig. 1:**
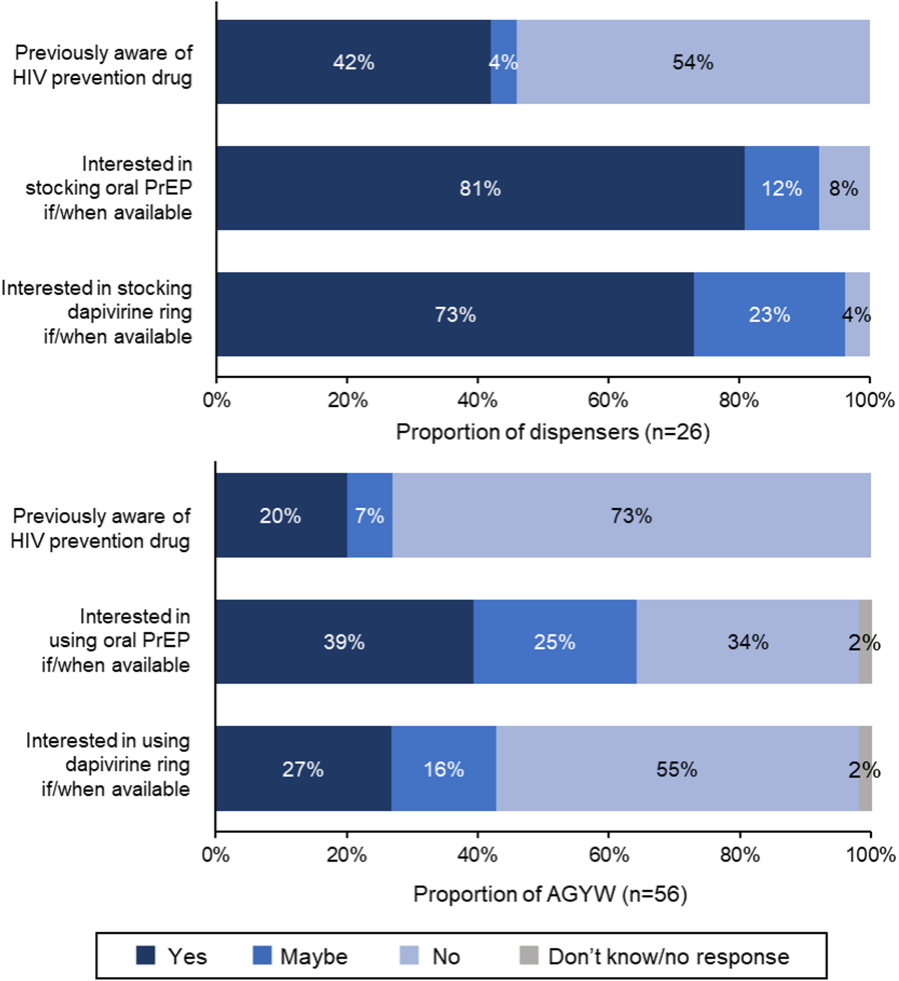
Dispenser (n = 26) and AGYW (n = 56) awareness of pre-exposure prophylaxis for HIV prevention and interest in stocking/using oral PrEP and the dapivirine ring if available in Shinyanga, Tanzania, 2019. *AGYW* Adolescent girls and young women

Of 47 drug shops sampled, 27 could be contacted and met eligibility criteria; of these, 26 consented to participate. Among dispensers, the median age was 42 years (interquartile range [IQR] 29–55) and 77% were female (Table 1); most (62%) owned the shop. Overall, 42% of dispensers (95% CI 23.4–63.1%) had heard of a medication for HIV prevention (Fig. 1). After receiving verbal information on each product, 92% of dispensers (95% CI 74.9–99.1%) reported some interest in stocking oral PrEP, and 96% (95% CI 80.4–99.9%) reported some interest in the dapivirine ring. All dispensers reported some interest in stocking at least one of the two products, and most reported that they believed their customers would be interested in oral PrEP (81%; 95% CI 60.6–93.4%) and the dapvirine ring (65%; 95% CI 44.3–82.9%). Most dispensers (85%; 95% CI 65.1–95.6%) reported they would provide oral PrEP to any AGYW who requested it.

**Table 1:** Sample characteristics of drug shop dispensers

Characteristics of dispensers	Dispensers (n = 26)^a^
Age in years, median (IQR)	42 (29–55)
Sex	
Female	20 (77%)
Male	6 (23%)
Highest education level	
Primary school	5 (19%)
Secondary school, some post-secondary education, or other certificate	13 (50%)
University degree or diploma course	8 (31%)
Position at shop	
Shop owner	16 (62%)
Shop staff	10 (38%)
Years working at a drug shop/pharmacy, median (IQR)	8.5 (5–17)
Shop location type	
Urban	20 (77%)
Peri-urban	6 (23%)
Total number of shop staff	
1	18 (69%)
2	3 (12%)
3+	5 (19%)
Estimated number of customers that day	
Total customers, median (IQR)	24 (16.5–40)
AGYW customers, median (IQR)	6 (3–10)

^a^All 26 dispensers reported interest in at least one form of PrEP (either oral PrEP or the dapivirine ring)

A total of 56 AGYW customers (80% of those approached) from 11 of the 26 shops agreed to participate. Among AGYW customers, the median age was 17 years (IQR 16–19); 21% (95% CI 11.6–34.4%) had completed secondary school and 61% (95% CI 46.8–73.5%) were still in school (Table 2). AGYW lived a median of 10 min away (IQR 5–20) by walking, bicycle, or motorcycle taxi from the shop where they were interviewed. About 29% of AGYW (95% CI 15.8–40.3%) were married or had a serious partner and 18% (95% CI 8.9–30.4%) had children. Most AGYW (77%; 95% CI 63.6–87.0%) did not have a job or way to earn money and about 20% (95% CI 10.2–32.4%) reported moderate to severe household food insecurity.

**Table 2:** Sample characteristics of adolescent girls and young women (AGYW) customers

Characteristics of AGYW customers	All AGYW (n = 56)	By interest in PrEP (either form)^a^		*p *value^b^
		Some interest (n = 39)	No interest (n = 16)	
Age in years, median (IQR)	17 (16–19)	17 (16–21)	16 (15.5–17)	0.053
Currently in school				0.072
Yes	34 (61%)	21 (54%)	13 (81%)	
No	22 (39%)	18 (46%)	3 (19%)	
Highest/current education level				0.438
Primary school or no level completed	7 (13%)	6 (15%)	1 (6%)	
Some secondary school	37 (66%)	24 (62%)	13 (81%)	
Secondary school	12 (21%)	9 (23%)	2 (12%)	
Have job or earn money				0.304
Yes	13 (23%)	11 (28%)	2 (12%)	
No	43 (77%)	28 (72%)	14 (88%)	
Relationship status				**0.047**
Single	27 (48%)	16 (41%)	11 (69%)	
Married or steady partner	16 (29%)	14 (36%)	1 (6%)	
Casual or multiple partner(s)	13 (23%)	9 (23%)	4 (25%)	
Have children				**0.046**
Yes	10 (18%)	9 (23%)	0 (0%)	
No	46 (82%)	30 (77%)	16 (100%)	
Household food insecurity				1.000
Little to no household hunger	45 (80%)	31 (79%)	13 (81%)	
Moderate/severe household hunger	11 (20%)	8 (21%)	3 (19%)	
Distance to shop in minutes, median (IQR)	10 (5–20)	12.5 (7.5–17.5)	10 (5–25)	0.896
Reason for shop visit				0.519
SRH product(s)	14 (25%)	9 (23%)	5 (31%)	
Other	42 (75%)	30 (77%)	11 (69%)	
Previously aware of HIV prevention drugs				0.478
Yes	11 (20%)	9 (23%)	2 (13%)	
No or unsure	45 (80%)	30 (77%)	14 (88%)	

^a^AGYW characteristics are stratified by some interest or no interest in at least one form of PrEP (either oral PrEP or the dapivirine ring). One AGYW customer responded “don’t know/no response” to the questions about interest in using oral PrEP and the dapivirine ring

^b^For categorical variables, p values from Fisher exact tests are presented. For continuous variables, p values from Kruskal–Wallis tests are presented. Significant associations (at α = 0.05) are shown in bold

Only 20% of AGYW (95% CI 10.2–32.4%) had heard of a medication to prevent HIV, yet 70% (95% CI 55.9–81.2%) expressed some interest in using at least one of the two forms for PrEP after receiving information about the products. More specifically, 64% (95% CI 50.3–76.6%) and 43% (95% CI 29.7–56.8%) of AGYW reported some interest in using oral PrEP and the dapivirine ring, respectively. We observed higher interest in PrEP among AGYW who were partnered and had children. More specifically, 79% (95% CI 60.3–92.0%) of AGYW with partners were interested in PrEP compared to 59% (95% CI 38.8–77.6%) of single AGYW, and all AGYW with children (95% CI 66.4–100%) were interested in PrEP compared to 65% (95% CI 49.8–78.6%) of AGYW without children. Otherwise, we did not observe any significant bivariate associations between measured AGYW characteristics and interest in using PrEP.

## Discussion

This study evaluated PrEP awareness and interest in PrEP among dispensers and AGYW customers at 26 drug shops. We found low prior awareness of PrEP among both groups. After receiving basic information on oral PrEP and the dapivirine ring, however, almost all dispensers reported some interest in stocking both products and indicated they would sell oral PrEP to AGYW. In comparison, a study of healthcare providers in Tanzania found that only 61% were willing to prescribe PrEP to AGYW [32]. However, shops in our sample were participating in a parent study focused on HIV prevention and SRH among AGYW, which likely influenced dispensers’ responses. Furthermore, willingness to provide PrEP to AGYW in the aforementioned study of facility-based providers was defined as willing to prescribe for all five AGYW sub-groups listed in the survey (e.g., AGYW in sero-discordant relationships, AGYW who exchange sex for money etc.), whereas we asked about willingness to provide PrEP to AGYW in general.

Compared to dispensers, we found lower, yet still considerable, levels of interest in PrEP among AGYW. Comparable interest levels have been found among AGYW elsewhere in sub-Saharan Africa, who—like AGYW in this study—have also reported lower interest in the dapivirine ring than in oral PrEP [33–35]. This may be partially explained by the lack of familiarity with vaginal ring contraceptives in the region [34]. Because the dapivirine ring is not currently available in Tanzania, reported interest in the ring is hypothetical. Nevertheless, this work is both timely and necessary to understand appropriate implementation models for the ring’s eventual rollout, the timeline of which has been accelerated by the European Medicines Agency’s recent positive benefit-risk opinion on the ring [36]. The values and preferences of women, in conjunction with safety and efficacy evidence, will be of important consideration as regulatory decisions and guidelines for the ring are developed [36].

Our findings make several key contributions. While ADDOs have been utilized to improve access to many pharmaceutical services in Tanzania and other low- and middle-income countries [25, 26], this is the first study, to our knowledge, that explores the potential of drug shops to expand PrEP access in these settings. Shinyanga, in particular, is a valuable research setting, as ADDO-based provision of PrEP would likely have greater impact in rural areas with limited healthcare facilities and many other access barriers. Although AGYW in this study had access to HIV self-testing at participating shops through the parent study, only 20% were aware of medication to prevent HIV. As HIV self-test kits are introduced to new markets, HIV prevention efforts could be amplified if complemented with additional, accessible prevention services. Adding HIV testing and PrEP services to drug shops would be a logical fit, creating one-stop-shops to meet AGYW’s SRH needs.

This study has important limitations. Small sample sizes may have limited the power needed to detect associations with PrEP interest. Our sample included AGYW from only 11 of the 26 shops, which may be explained by low AGYW patronage at the remaining shops. Additionally, participating dispensers had consented to our parent study focused on AGYW SRH, which may have influenced their responses. However, the dispenser survey was administered at enrollment, prior to implementation of the intervention, thus limiting potential effects of the intervention on our findings. Nevertheless, if PrEP is to be sold at drug shops, it would be desirable to seek out ‘AGYW-friendly’ dispensers. HIV-related stigma and reliance on interviewer-administered questionnaires may have also influenced verbally self-reported PrEP awareness and interest levels. Finally, we did not collect data on AGYW’s HIV status or whether they were sexually active, which may have provided insight into their actual and perceived HIV risk and subsequent interest in PrEP.

## Conclusions

Recent studies have demonstrated promising models of private-sector PrEP delivery, yet there is limited research exploring this potential in low- and middle-income countries with high HIV prevalence [23, 37]. Our findings suggest the potential for drug shops to deliver PrEP to high-priority populations in Tanzania. Additional work is needed to assess the feasibility and effectiveness of this model and determine the role of drug shops in PrEP provision within the larger healthcare system. While we focused on oral PrEP and the dapivirine ring, drug-shop-based models could be extended to other forthcoming PrEP formulations (i.e., long-acting injectable PrEP, particularly if sold for self-administration). Additional training for dispensers would be needed to ensure PrEP literacy and address stigma around HIV and AGYW sexuality while complementary measures to increase consumer awareness of PrEP are developed. Despite these implementation challenges, privately-owned drug shops are uniquely positioned to reach AGYW and offer accessible, community-based HIV prevention services.

## Data Availability

The datasets used and/or analyzed during the current study are available from the corresponding author on reasonable request.

## Abbreviations

ADDO: Accredited drug dispensing outlet
AGYW: Adolescent girls and young women
PrEP: Pre-exposure prophylaxis
SRH: Sexual and reproductive health
